# The Effect of a Nature-Based Gel on Gingival Inflammation and the Proteomic Profile of Crevicular Fluid: A Randomized Clinical Trial

**DOI:** 10.3390/gels10120772

**Published:** 2024-11-27

**Authors:** Luciene Cristina Figueiredo, Bruno Bueno-Silva, Giovanna Denúncio, Nathalia Freitas Figueiredo, Daniele Ferreira da Cruz, Jamil A. Shibli, Maria Helena R. Borges, Valentim A. R. Barão, Doron Haim, Thabet Asbi, João Gabriel S. Souza

**Affiliations:** 1 Dental Research Division, Guarulhos University, Guarulhos 07023-070, SP, Brazil; 2 Department of Biosciences, Piracicaba Dental School, Universidade Estadual de Campinas (UNICAMP), Piracicaba 13414-903, SP, Brazil; 3 Department of Prosthodontics and Periodontology, Piracicaba Dental School, Universidade Estadual de Campinas (UNICAMP), Piracicaba 13414-903, SP, Brazil; 4 Maccabi-Dent Research Department, Maccabi Healthcare Fund, Tel-Aviv 6801298, Israel; 5 Department of Periodontology and Implant Dentistry, Rambam Health Care Campus, Haifa 3525408, Israel

**Keywords:** toothpaste, gingivitis, proteomic

## Abstract

Evidence has shown the clear positive effects of nature-based products on biofilm control and improved gingival health. However, most studies have used in vitro models, have tested single natural components, or have not evaluated proteomic changes after treatment. This double-blind, parallel, randomized, and controlled clinical trial evaluated the benefits of a nature-based gel in controlling gingival inflammation and its effects on the proteomic gingival crevicular fluid (GCF) profile. Gingivitis patients were distributed into the following groups: (1) nature-based gel containing propolis, aloe vera, green tea, cranberry, and calendula (n = 10); (2) control—conventional toothpaste (n = 10). GCF was collected and evaluated by means of liquid chromatography coupled with tandem mass spectrometry (LC–MS/MS). At 3 months, the groups showed similar clinical benefits (*p* < 0.05). A total of 480 proteins were identified across all groups. In a pooled comparison of both groups at both time points, exclusive proteins were identified in the nature-based gel (78) and the control (21) groups. The exclusive proteins identified for the toothpaste mainly acted in wound healing, and those for the nature-based gel mainly acted on immune system processes. The nature-based gel achieved similar clinical outcomes to conventional toothpaste. However, the nature-based gel markedly changed the proteomic profile of GCF after treatment, showing a profile associated with a host response.

## 1. Introduction

Polymicrobial dysbiotic oral biofilms are the main etiologic factor triggering inflammatory chronic diseases on tooth-supporting tissues, known as periodontal diseases [1]. These prevalent conditions affect millions of people worldwide, showing increasing rates with age and negatively impacting people’s lives [2]. The biofilm’s biomass and its microbial composition act as chemical and physical “stress” factors on tooth-supporting tissues, triggering inflammatory responses and subsequent tissue damage [3]. This initial process is characterized by clinical gingival inflammation, known as gingivitis, which often progresses in severity and tissue damage [4]. Since it is a multifactorial condition, different risk, genetic, and systemic factors, as well as the intensity and duration of microbial challenges and host susceptibility, may promote the destruction of periodontal supporting tissue [5]. Oral hygiene procedures, such as toothbrushing and other mechanical cleansing methods, are effective in controlling biofilm accumulation and achieving gingival health [6]. Different antimicrobial agents and adjunctive therapies have been tested to control oral biofilm accumulation and reduce gingival inflammation [7], and the composition of toothpaste also plays an important role in achieving these outcomes [6].

Previous evidence suggests that specific chemical agents in toothpaste may present enhanced benefits in controlling gingival inflammation [6]. In fact, toothpaste composition may also affect local molecular parameters, such as microbial pathways at the metatranscriptomic level and the levels of pro-inflammatory components in gingival crevicular fluid (GCF) [8]. These new molecular and omics approaches provide new insights into the modulation of the abilities of new products and explain the effects of different agents on oral sites. However, although the proteomic profile is responsible for mediating molecular functions and biological outcomes, it has not been tested in new toothpastes, especially at the GCF level [9]. An understanding of the proteins involved in health and disease states provides significant potential in identifying new biomarkers, given the close relationship between molecular activity and biological/clinical outcomes [10].

In this sense, changes in toothpaste formulation should consider not only the clinical evaluation of gingival conditions and biofilm control but also the local molecular parameters that drive one toward a health-associated state. Incorporating new agents to enhance oral biofilm control and address its pathogenicity is a logical development in improving clinical outcomes [11]. Although traditional components have been used in most commercially available toothpastes, new formulations have been explored as agents, such as nature-based compounds. Thus, over the past decade, there has been increasing interest in nature-based products for oral health [12]. In the United States of America (USA), a study identified that 35% of the investigated population reported using some form of herbal medicine [13]. Furthermore, a high proportion of new drugs approved by the Food and Drug Administration (FDA) are natural or naturally derived products [14]. These products have shown high antimicrobial abilities, combined with additional benefits, such as anti-inflammatory and antioxidant properties [12]. Evidence shows the clear positive effects of nature-based products on biofilm control and improved gingival health, but most of this evidence comes from in vitro models [12]. Moreover, most studies have only tested products with single natural components [12], and combinations in a single product have not been widely explored, especially in terms of proteomic changes after treatments.

Therefore, this clinical trial aims to evaluate and compare the clinical benefits of a nature-based gel in controlling gingivitis and its effects on the proteomic GCF profile compared with a conventional toothpaste.

## 2. Results and Discussion

### 2.1. Clinical Outcomes

Natural products have shown promising antimicrobial effects against periopathogens, but most evidence has emerged from in vitro models [15]. These outcomes demonstrate the anti-inflammatory and antioxidant abilities of these agents, making them effective compounds in the manufacturing of nature-based products to control gingival inflammation. It is important to note that most of the clinical evidence has come from tests of nature-based products for oral biofilm control using mouthrinses as the vehicle [12]. This clinical study included 20 subjects (DESPLAC^®^ group: 8 women and 2 men, 30–47 years of age; and Oral-B group: 7 women and 3 men, 31–48 years of age). There were no dropouts during the experimental period. All subjects who finished the study reported full adherence to the prescribed oral hygiene protocol. Throughout the study, no adverse effects on the soft or hard tissues of the oral cavity were detected by the examiner or reported by the subjects. Table 1 presents the mean (± SD) of full-mouth clinical parameters evaluated at baseline and 3 months after using the products. At the beginning, there were no clinical differences between the groups for any of the evaluated parameters (*p* > 0.05), demonstrating the homogeneity of the sample groups. At 3 months, no statistically significant differences were observed between the two groups (*p* > 0.05); however, a reduction in the gingival index was noted in both the nature-based gel and control toothpaste groups. Moreover, when evaluating the proportion of each score on the plaque index, there was a clear reduction trend after 3 months, with an approximately 20% increase in the number of sites/teeth categorized with a score of 0 on the plaque index (Table S1). Bleeding on probing, expressed as the percentage of sites bleeding, also did not show any differences between the groups or time points (Table S2). Therefore, the clinical findings showed similar outcomes for both tested products (nature-based gel and control toothpaste) after 3 months, with both being effective in reducing gingival inflammation.

DESPLAC^®^ gel (Premium Oral Gel) includes a combination of different natural agents in its formula: propolis, aloe vera, green tea, cranberry, and calendula. Although the individual antimicrobial, anti-inflammatory, and/or antioxidant benefits of these natural agents are well known, their combination in a single product has not been widely explored [16,17,18,19]. Previous in vitro studies have explored the antimicrobial ability of DESPLAC^®^ in a multispecies biofilm model [16,17,18,19]. The results demonstrated its potential to reduce bacterial metabolic activity and the levels of two important periopathogens: *Tannerella forsythia* and *Porphyromonas gingivalis* [20,21]. In our clinical setting with gingivitis patients, the nature-based gel was clinically effective, controlling gingival inflammation over 3 months of use. However, the clinical outcomes of the two tested products were similar, with the nature-based gel showing no additional benefit compared with conventional toothpaste. In fact, a systematic review demonstrated that mechanically removing biofilm through toothbrushing is more important than the presence of toothpaste [22]. However, toothpaste may contain other important chemical agents crucial for oral health, such as fluoride, which is well known for its ability to control dental caries but is absent in the DESPLAC^®^ product [23]. Importantly, clinical trials like the one conducted here, comparing different gel/toothpaste formulations for oral biofilm and gingival inflammation control, may be helpful in further developing clinical guidelines, particularly regarding the use of nature-based products for periodontal health and respecting patients’ preferences and characteristics [12]. Moreover, although a 3-month follow-up period is commonly used in clinical trials to identify significant changes in gingival health recovery post-treatment, aligning with the gingival healing timeline and the recommended interval for maintenance visits, longer periods should be considered in future studies to evaluate the maintenance of outcomes [24,25]. Furthermore, the reduction in gingival inflammation was not accompanied by a reduced plaque index after 3 months. However, the plaque index was expressed as an average of scores, and when evaluating the proportion of each score, there was a clear improvement in plaque control, with an increased proportion of sites scoring 0 after 3 months (Supplementary Materials). The lack of differences in the average values for this outcome may be attributable to the sample size, necessitating further investigation.

### 2.2. Composition of Toothpaste Modulated the Proteomic Profile in GFC

**Figure 1:**
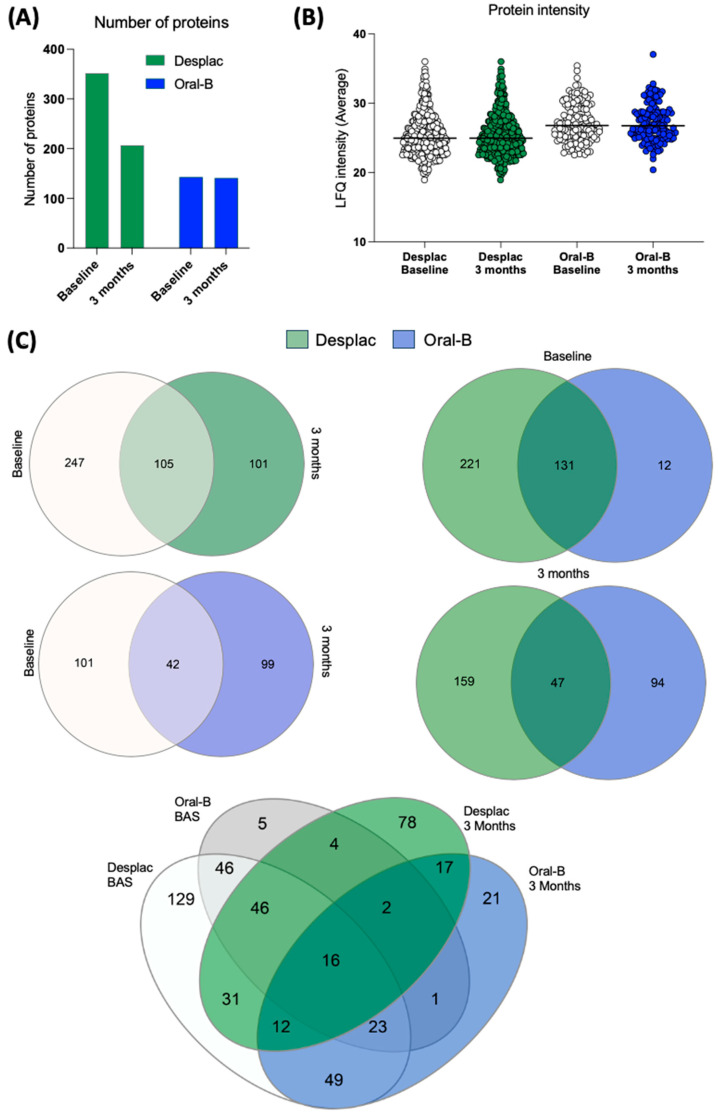
Proteomic profile of gingival crevicular fluid of patients treated with nature-based gel (DESPLAC^®^) or conventional toothpaste (Oral-B). (**A**) Total proteins identified for each group and time point (baseline and after 3 months). Proteomic profile was evaluated using liquid chromatography coupled with tandem mass spectrometry. (**B**) Average LFQ intensity of proteins identified for each group and time point. (**C**) Venn diagrams comparing the groups and time points.

**Figure 2:**
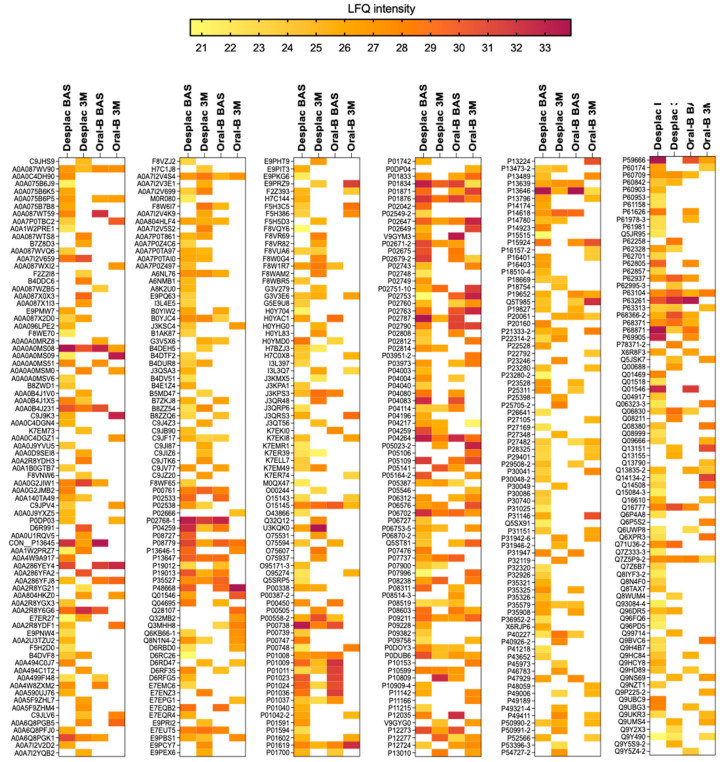
Proteomic profile of gingival crevicular fluid of patients treated with nature-based gel (DESPLAC^®^) or conventional toothpaste (Oral-B) at different time points (BAS—baseline; or 3M—3 months). Heatmap of LFQ intensity of proteins identified for each group and time point.

Our pioneering study explored the effect of a nature-based product on the GCF proteomic profile of gingivitis patients. A total of 480 proteins were identified across all groups at both time points. The conventional toothpaste group presented 143 proteins at baseline and 141 after 3 months (Figure 1A). For the nature-based gel group, 352 proteins were identified at baseline and 206 after 3 months (Figure 1A). Although the number of proteins somewhat decreased after 3 months, their intensities were similar for both groups, with no significant differences over time (Figure 1B). Importantly, both groups showed notable differences in the proteomic profile of GCF after 3 months compared with baseline (Figure 1C). Exclusive proteins were identified in the conventional toothpaste group (99 proteins) and the nature-based gel group (101 proteins) after 3 months within the same-group comparison. When comparing the two groups at each time point, our findings showed a lower level of similarity, with approximately 36% of proteins being shared at baseline and only 15.6% after 3 months (Figure 1C). In the pooled comparison of the groups at each time point, exclusive proteins were identified in both, indicating differences in proteomic profile according to time and treatment. These results demonstrate important differences in the proteomic profile of GCF during gingivitis treatment/control, which were also modulated by the toothpaste/gel applied. Despite differences in proteomic composition and the presence of exclusive proteins in each group, the heatmap shows that even among shared proteins, some differences in intensities were identified between both groups (Figure 2).

Periodontal disease progression markedly changes the proteomic profile of GCF [26]. Moreover, changes in GCF protein levels caused by inflammation and disease resolution have been reported [27]. However, an important level of overlap in proteomic profile between healthy individuals and those with periodontal disease has been found [28], as reported in our study comparing scores at baseline and after 3 months. Our findings show that the nature-based gel increased the number of proteins identified in GCF, and compared with conventional toothpaste, it showed only 15.6% similarity after 3 months. Importantly, the pooled comparison (including both groups and time points) revealed that specific proteins could be uniquely identified for each group: 78 proteins were exclusive to the nature-based gel group, while 21 proteins were unique to the conventional toothpaste group at 3 months. This shows that the composition of the products highly affected the proteomic composition of GCF after 3 months of use. Notably, GCF is a complex mixture with various components, and its composition is affected by factors such as aging and biofilm composition [29]. Although all patients were standardized in terms of clinical parameters, molecular factors may have contributed to increased protein content in the nature-based gel group at baseline. However, after 3 months, the groups showed close protein quantities, with differences primarily driven by composition, which should be further investigated at the screening stage. Moreover, there was a clear change in the proteomic profile within the nature-based gel group when comparing the baseline and the 3-month follow-up, suggesting that the identified proteomes are a result of the treatment rather than the initial composition.

**Figure 3:**
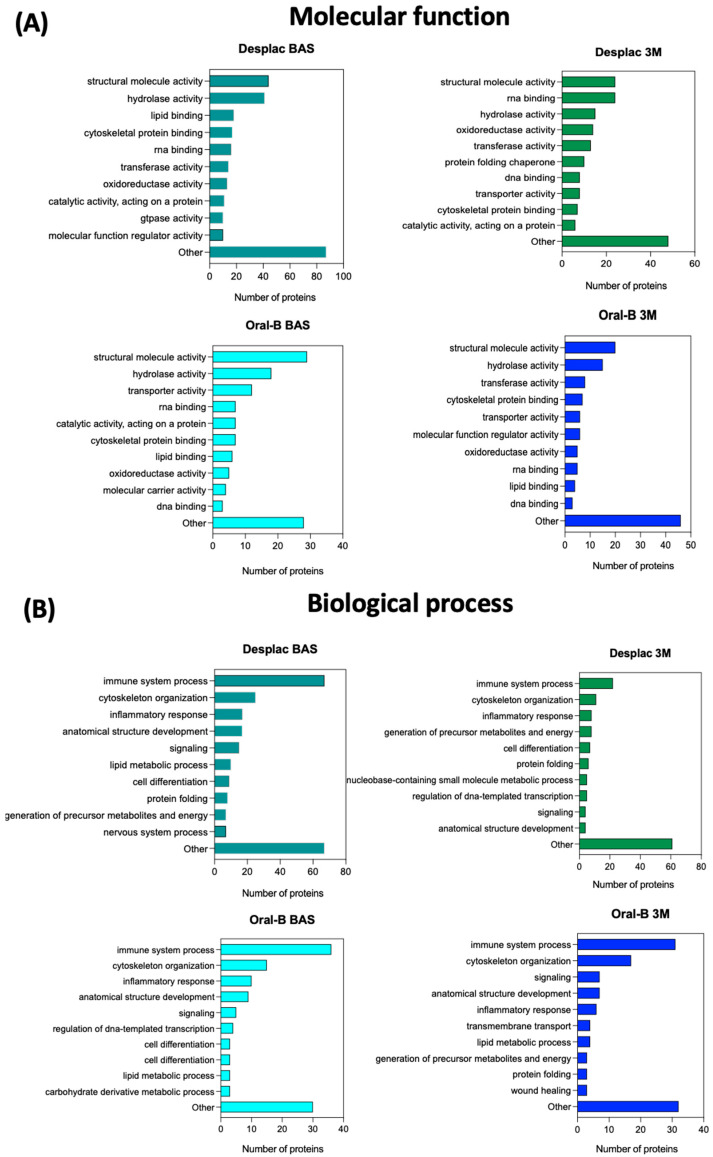
Molecular functions and biological processes mediated by the proteins identified in each group and at each time point. Top 10 molecular functions (**A**) and biological processes (**B**) with the highest number of proteins adsorbed onto each substrate. Nature-based gel (DESPLAC^®^) or conventional toothpaste (Oral-B) at different time points (BA—baseline; or 3M—3 months).

The exclusive proteins identified for each group in the pooled comparison (all groups and time points—Figure 1C, bottom diagram) are described in Table 2. After 3 months, the exclusive proteins identified for the conventional toothpaste mainly acted in wound healing, the most common biological process identified. In contrast, for the nature-based gel group, the exclusive proteins identified after 3 months primarily mediated immune system processes (Table 2). Therefore, considering that each protein has a specific molecular function and mediates specific biological processes, the profile for all proteins (not only exclusive ones) identified in each group and at each time point was also explored (Figure 3A,B). Although there is a high level of similarity between the groups at both time points in terms of the top 10 most common molecular functions and biological processes related to the identified proteins, some slight differences were observed. The inflammatory response process was among the top three biological processes for the nature-based gel group after 3 months but not for the conventional toothpaste group. Furthermore, cell differentiation was a common biological process identified for the nature-based gel but not for the conventional toothpaste. However, wound healing was only found among the common biological processes for the conventional toothpaste (Figure 3B). Therefore, the differences in proteomic profile led to slight differences in the main molecular functions and biological processes mediated by the identified proteins. The immune response process plays a key role in restoring homeostasis in injured tissues [30]. Therefore, the nature-based product and its composition may enhance the host response, and some of its components—such as propolis, with its well-recognized anti-inflammatory properties—may also contribute to faster inflammation resolution [30,31]. Although immune-response-related proteins were highly associated with the nature-based gel, clinical parameters, as measured by the gingival index, suggest the resolution of the inflammatory process, similar to what was observed with the conventional toothpaste, indicating no delay in the healing process for this group. Notably, the same protein can exhibit a wide range of molecular functions and be involved in different biological processes. While proteins may have primary functions, they can play roles in various processes. Moreover, the presence of a specific protein does not necessarily indicate a high concentration; some components of GCF may show similar levels immediately after therapy compared with the baseline but then exhibit reduced levels during the maintenance phase [32].

The exclusive proteins identified in each group also need to be explored. For example, the conventional toothpaste group showed the exclusive protein integrin beta-3 at 3 months, which is part of a protein group associated with stimulating growth factors, forming granulation tissue, and modulating inflammatory processes [33]. The nature-based gel group also presented an important exclusive proteomic profile at 3 months, including the presence of Peroxiredoxin-2, which plays a role in immune system processes and has well-recognized antioxidant abilities [34]. These findings may provide important information on clinical changes at the molecular level and reveal new biomarkers for testing the effectiveness of therapeutic products or disease resolution.

Although these findings are promising in demonstrating the effectiveness of a nature-based product in controlling biofilm accumulation and gingival inflammation—and although 3 months of evaluation is commonly used for this type of trial [12]—further clinical trials should evaluate the maintenance of outcomes over longer periods. Moreover, the use of high-throughput techniques focusing on the oral microbiome should also be further explored and aligned with evaluating immune response pathways. Furthermore, the sample size used here may have reduced the chance of identifying differences between the groups, and further trials should consider larger samples. Clinical trials testing these new nature-based products are essential to elucidating the clinical efficacy of these formulations. However, patient profiles, the occurrence of other oral diseases, adverse effects, and the therapeutic protocols applied need to be considered before recommending these products. Moreover, patients need to be well informed about oral hygiene procedures and the importance of mechanical biofilm control through toothbrushing, regardless of toothpaste formulation.

## 3. Conclusions

We demonstrated that a nature-based gel achieved similar clinical outcomes to a conventional toothpaste in controlling gingival inflammation among gingivitis patients. However, the nature-based gel markedly changed the proteomic profile of GCF after treatment compared with the conventional toothpaste, increasing the number of proteins and showing a profile associated with a host response.

## 4. Materials and Methods

### 4.1. Study Design

This study protocol was developed following SPIRIT (Standard Protocol Items: Recommendations for Interventional Studies), and the manuscript was prepared according to the CONSORT checklist [35]. This was a double-blind, parallel, randomized, and controlled study. All subjects received and signed a form of informed consent. The study protocol was approved by the local Ethics Committee (IRB approval #51781621.9.0000.5506), and the clinical trial was registered (RBR-7dz5y9x—REBEC platform).

### 4.2. Treatment Groups

Using a table of equiprobable numbers, all selected individuals were randomly distributed to the following treatment groups: (1) test group (n = 10): nature-based gel (DESPLAC^®^, São Paulo, São Paulo, Brazil)—applied 2x/day during toothbrushing in the morning and evening; (2) control group (n = 10): Oral-B ProGengiva (Cincinnati, OH, EUA)—applied 2x/day during toothbrushing in the morning and evening. The DESPLAC^®^ product (Premium Oral Gel) is the only toothpaste available on the Brazilian national market that includes a combination of natural agents in its formula: propolis, aloe vera, green tea, cranberry, and calendula. Considering the outcomes found for the average and standard deviation values of the nature-based gel group’s gingival index at baseline and after 90 days, the sample power was estimated using an α of 5% and an effect size of 1.23, resulting in a power of 90%.

### 4.3. Subject Population and Inclusion/Exclusion Criteria

Participants were selected from the population referred to the Dental Clinic of Guarulhos University (Guarulhos, SP, Brazil). The inclusion criteria were as follows: availability for the duration of the study; at least 15 natural teeth with minimal tooth restorations; good general health or health well controlled under a physician’s care; aged 18–65 years; and an average initial gingivitis index of at least 1.5 [36]. The exclusion criteria were as follows: a medical condition requiring premedication before dental visits/procedures; use of any medication that may affect salivary flow; xerostomia; carious lesions; sites with probing depths of >4 mm; use of orthodontic appliances; use of antibiotics within 6 months before or during the study; use of any over-the-counter medications that would affect the results, other than analgesics (i.e., aspirin, ibuprofen, acetaminophen, or naproxen) at the time of informed consent; pregnant or breastfeeding individuals; immune-compromised individuals; history of allergies to oral-care/personal-care consumer products or their ingredients; and a history of alcohol abuse, smoking, or drug abuse.

### 4.4. Procedures

All participants received the same oral hygiene instructions in terms of products to be used, a toothbrush with soft bristles, and the respective tube of toothpaste according to their assigned group. The study coordinator, who was uninvolved in the clinical evaluations, distributed the oral hygiene products to the participants. Allocation concealment was achieved using numbered, opaque, and sealed envelopes handled by the study coordinator. All products were stored in a sealed bag to eliminate any differences in product aesthetics and packaging between the study groups. Label information included a study group code, instructions for at-home use, and safety information (including emergency contact details). Participants were instructed to apply 1 cm of toothpaste to the brush and brush their teeth for 2 min twice a day (morning and evening) and to return their assigned products to the study site before receiving new products. They were instructed to follow these oral hygiene regimens for 90 days and to exclusively use the provided products. There were no specific instructions related to toothbrushing technique. They were not instructed to use dental floss to avoid introducing additional factors that could affect outcomes related to the toothpaste/gel composition and use. At the end of the 90 days, all participants returned to the dental clinic for a clinical evaluation and for the collection of gingival crevicular fluid samples. They were also instructed to refrain from performing any oral hygiene procedures for 4 h before the clinical evaluation at 90 days. The clinical evaluations and sample collections were conducted by calibrated examiners. Calibration was conducted according to a previously established protocol for periodontal studies [37]. The calibration phase was conducted before the study began, with examiners assessing specific clinical conditions in patients after familiarizing themselves with the protocol measures. At the first clinical appointment, an examiner assessed up to 42 sites per patient, followed by a second examiner who repeated the measurements in the same quadrant during the same appointment. Seven days later, at the second appointment, one of the original examiners and a third examiner repeated the measurements. The standard error of measurement was calculated, with intra-examiner variability at 0.26 mm and 0.28 mm for probing depth and clinical attachment level, respectively; inter-examiner values were 0.25 mm and 0.29 mm for probing depth and clinical attachment level, respectively, showing adequate reproducibility for periodontal evaluations. For categorical variables, such as plaque index and bleeding on probing, the level of agreement was estimated using Kappa, showing a 91% agreement rate.

### 4.5. Clinical Evaluation

At baseline and at 90 days, the presence or absence of visible plaque, marginal bleeding, and bleeding on probing, as well as probing depth and clinical attachment level, were assessed at six sites per tooth (excluding third molars) using a manual periodontal probe (North Carolina—Hu-Friedy, Chicago, IL, USA). Plaque accumulation and gingival inflammation were assessed for each tooth using standardized methods: the gingival index [36] and plaque index [38]. Each tooth was categorized by score, and an average score was calculated. The percentage of teeth assigned to each score was also calculated. Bleeding on probing was expressed as the average number of bleeding sites [39]. Probing depth and clinical attachment level were estimated in millimeters and expressed as an average.

### 4.6. Monitoring Compliance and Adverse Events

At the end of the study, participants were asked to return to the dental clinic and bring their tube(s) of toothpaste (empty or not), which were checked for any remaining toothpaste. The oral soft tissues of all subjects were evaluated during the clinical assessment at 90 days, and subjects were queried about any self-perceived side effects.

### 4.7. Gingival Crevicular Fluid (GCF) Sampling

The same GCF sampling procedure was performed for all patients. GCF was collected approximately one week after the clinical evaluation to avoid changes in the GCF due to blood contamination. Two non-adjacent shallow sites with a PD and CAL of ≤3 mm were randomly selected per patient. Supragingival biofilm was removed, and the sites were isolated and dried to avoid saliva contamination. GCF was sampled by inserting a standard absorbent paper cone approximately 2 mm into the sulcus/pocket for 60 s. After 20 s, a second absorbent paper cone was inserted in the same site for another 60 s. The two paper cones from the same site were pooled in a single dry microcentrifuge tube and stored at −80 °C. Strips contaminated with blood were discarded.

### 4.8. Protein Extraction

The absorbent papers containing the samples were placed in tubes with 150 μL of protein extraction solution (58% acetonitrile, 40% purified water, and 2% acetic acid). Each tube was agitated for 1 min, and the extraction procedure was performed in duplicate for each sample. After extraction, the solutions were pooled by group to increase protein concentration to a detectable level for the technique. The samples were then dried for 2 h, resuspended in ammonium bicarbonate (50 mM; pH 7.8), and dried again for an additional 1 h.

### 4.9. Liquid Chromatography Coupled with Tandem Mass Spectrometry

The proteomic profile was evaluated using liquid chromatography coupled with tandem mass spectrometry (LC–MS/MS), following a previous protocol [40,41,42]. Pooled samples were resuspended in urea (8 M). The Bradford method was used to quantify the total protein content. Then, the proteins were reduced, alkylated, digested with trypsin (1:50 w w-1), and subjected to LC–MS/MS. Samples were dried in a vacuum concentrator and immersed in 22.5 µL of 0.1% formic acid. An aliquot of 2 µL (0.88 µg) was analyzed on an LTQ Orbitrap Velos mass spectrometer (Thermo Fisher Scientific, Waltham, MA, USA) connected to an EASY-nLC system (Proxeon Biosystem, West Palm Beach, FL, USA) through a Proxeon nanoelectrospray ion source. Peptides were separated by applying a 2–90% acetonitrile gradient in 0.1% formic acid in an analytical PicoFrit Column (20 cm × ID75 µm, 5 µm particle size) (New Objective Inc., Woburn, MA, USA) over 80 min [43]. The instrument methods were set up in the data-dependent acquisition mode. After accumulation to a target value of 1 × 106, full-scan MS spectra (*m*/*z* 300–1600) were made from an Orbitrap analyzer with a resolution of r = 60,000. A thousand (1000) counts was the signal threshold for triggering an MS/MS event. A size list of 500, a duration of 60 s, and a repeat count of 1 were enabled for dynamic exclusion.

Peptide sequences acquired were identified using MaxQuant (v.1.3.0.3—Martinsried, Munich, Germany), and MS/MS spectra were searched against the Human UniProt database. A maximum of 1%FDR was set for both protein and peptide identification. Protein quantification was performed using the LFQ algorithm implemented in MaxQuant, with a minimum ratio count of 2 and a window of 2 min for matching between runs. The list of peptides identified was filtered by a minimum 0.75 localization probability of containing at least one peptide with Perseus v.1.5. Reverse and contaminant entries were excluded from further analysis.

Exclusive proteins identified in each group were depicted using Venn diagrams constructed using a free web-based tool [44]. The name, molecular function, and biological process of each protein were checked using the UniProt database. Protein ID was used to search manually on UniProt for all information. Heatmaps were constructed using GraphPad Prism (version 8.0.0 for Windows, GraphPad Software, San Diego, CA, USA) to show LFQ intensity (protein intensity/expression) for each group and time.

### 4.10. Statistics

Prism (GraphPad) 8.0 and SPSS (20.0) were used to generate graphs and perform statistical analysis. Group comparisons, when necessary, were conducted using the *t*-test or repeated measures ANOVA (Tukey’s test). A significance level of 5% was adopted.

## Figures and Tables

**Table 1:** Full-mouth clinical parameter evaluation of the study population at baseline and after 3 months. Average (± SD).

		Groups (Average and SD)	
Parameters	Time Point	DESPLAC^®^ (n = 10)	ORALB (n = 10)
			
Plaque index	Baseline	0.78 ± 0.59 ^a^	0.82 ± 0.61 ^a^
	3 months	0.82 ± 0.6 ^a^	0.86 ± 0.72 ^a^
			
Gingival index	Baseline	1.52 + 0.57 ^aA^	1.50 + 0.57 ^aA^
	3 months	0.81 ± 0.58 ^aB^	0.73 ± 0.53 ^aB^
			
Probing depth (mm)	Baseline	2.34 ± 0.62 ^a^	2.33 ± 0.60 ^a^
	3 months	2.33 ± 0.60 ^a^	2.28 ± 0.67 ^a^
			
Clinical attachment level (mm)	Baseline	2.41 ± 0.65 ^a^	2.39 ± 0.67 ^a^
	3 months	2.39 ± 0.67 ^a^	2.37 ± 0.68 ^a^
			
Bleeding on probing	Baseline	0.28 ± 0.45 ^a^	0.34 ± 0.47 ^a^
	3 months	0.34 ± 0.47 ^a^	0.20 ± 0.40 ^a^
			

Plaque index and gingival index are expressed as averages of scores. Bleeding on probing is expressed as the average number of affected sites. The significance of differences in clinical parameters between groups at baseline and 3 months were compared using the *t*-test (*p* > 0.05—the same lowercase letters in the lines indicate no statistical differences). Different capital letters in the columns indicate significant differences over time based on repeated measures ANOVA (Tukey’s test, *p* < 0.05). SD—standard deviation.

**Table 2:** Protein ID, name, molecular function, and biological process of the exclusive proteins in each group and at each time when comparing the four groups’ proteomic profiles (data extracted from the UniProt database).

Protein ID	Name	Molecular Function	Biological Process
**Oral-B—Baseline**			
A0A087WZB5	Parvin beta	cytoskeletal protein binding	cytoskeleton organization
A0A0A0MRZ8	Immunoglobulin kappa variable 3D-11		immune system process
K7EKI0	Envoplakin	structural molecule activity	cytoskeleton organization
P08514-3	Integrin alpha-IIb	molecular adaptor activity	immune system process
Q9Y5Z4-2	Heme-binding protein 2		
**Oral-B—3 months**			
A0A087WXI2	Fc gamma binding protein		
A0A0C4DGZ1			
P02666	Beta-casein	molecular function regulator activity	inflammatory response
Q28107	Coagulation factor V	molecular adaptor activity	circulatory system process
Q32MB2	Keratin, type II cytoskeletal 73	structural molecule activity	cytoskeleton organization
E7EPG1	Multimerin 1		
K7ER74	Apolipoprotein C-II	molecular function regulator activity	lipid metabolic process
P0DP04	Immunoglobulin heavy variable 3-43D		immune system process
P03951-2	Coagulation factor XI	catalytic activity, acting on a protein	wound healing
P05106	Integrin beta-3	catalytic activity, acting on a protein	wound healing
P05546	Heparin cofactor 2	molecular function regulator activity	wound healing
P10153	Non-secretory ribonuclease	catalytic activity, acting on rna	defense response to other organism
P11166	Solute carrier family 2, facilitated glucose transporter member 1	transporter activity	transmembrane transport
P13224	Platelet glycoprotein Ib beta chain	molecular transducer activity	wound healing
P16157-2	Ankyrin-1	structural molecule activity	vesicle-mediated transport
P22792	Carboxypeptidase N subunit 2	catalytic activity	
P27105	Stomatin	molecular function regulator activity	transmembrane transport
P48059	LIM and senescent cell antigen-like-containing domain protein 1	gtpase activity	cell adhesion
Q13790	Apolipoprotein F	transporter activity	lipid metabolic process
Q6P5S2	Protein LEG1 homolog		
Q9P225-2	Dynein axonemal heavy chain 2	cytoskeletal motor activity	cell motility
**Desplac—Baseline**			
A0A0C4DH90	Immunoglobulin kappa variable 3/OR2-268 (non-functional)		immune system process
A0A075B6J9	Immunoglobulin lambda variable 2–18		immune system process
A0A075B6K5	Immunoglobulin lambda variable 3–9		immune system process
A0A075B7B8	Immunoglobulin heavy variable 3/OR16-12 (non-functional)		immune system process
A0A1W2PRE1	Guanine nucleotide-binding protein G(o) subunit alpha	gtpase activity	signaling
A0A087WVQ6	Clathrin heavy chain	structural molecule activity	intracellular protein transport
E9PMW7	Eukaryotic translation elongation factor 1 delta		
A0A096LPE2	SAA2-SAA4 readthrough		inflammatory response
F8WE70	Serpin family B member 13	hydrolase activity	
A0A0A0MSV6	Complement C1q B chain		
B8ZWD1	Acyl-CoA-binding protein	lipid binding	lipid metabolic process
A0A0C4DGN4	Zymogen granule protein 16B		
A0A0J9YVU5	Immunoglobulin heavy variable 2–70		immune system process
A0A1B0GTB7	Spectrin alpha, non-erythrocytic 1		
A0A0G2JMB2	Immunoglobulin heavy constant alpha 2		immune system process
A0A0J9YXZ5	IQ motif containing GTPase activating protein	molecular function regulator activity	cytoskeleton organization
A0A4W9A917	Immunoglobulin heavy constant gamma 3		immune system process
A0A2R8YGX3	Tropomyosin 4	cytoskeletal protein binding	cytoskeleton organization
E9PNW4	CD59 glycoprotein		immune system process
A0A2U3TZU2	Glucose-6-phosphate isomerase	isomerase activity	nucleobase-containing small molecule metabolic process
F5H2D0	complement subcomponent C1r	hydrolase activity	immune system process
A0A494C0J7	Transglutaminase-like domain-containing protein	transferase activity	
A0A590UJ76	Deleted in malignant brain tumors 1 protein		cell differentiation
A0A6Q8PFJ0	Lamin A/C	structural molecule activity	cytoskeleton organization
A0A7I2V2D2	Serpin family G member 1	molecular function regulator activity	
F8VZJ2	Nascent polypeptide associated complex subunit alpha		
M0R080	DnaJ homolog subfamily B member 1	molecular adaptor activity	regulation of dna-templated transcription
A0A7P0Z4C6	Transitional endoplasmic reticulum ATPase		
A0A7P0Z497	Peptidyl-prolyl cis-trans isomerase	catalytic activity, acting on a protein	protein folding
A6NMB1	Sialic acid-binding Ig-like lectin 16		defense response to other organism
A8K2U0	Alpha-2-macroglobulin-like protein 1	molecular function regulator activity	
E9PQ63	Carbonyl reductase 1	oxidoreductase activity	
I3L4E5	Aldehyde dehydrogenase, dimeric NADP-preferring	oxidoreductase activity	
B1AK87	F-actin-capping protein subunit beta	cytoskeletal protein binding	cytoskeleton organization
B4DTF2	Annexin	lipid binding	vesicle-mediated transport
J3QSA3	Ubiquitin B	protein tag activity	protein catabolic process
B4DV51	GTP-binding nuclear protein Ran	gtpase activity	nucleocytoplasmic transport
B4E1Z4	Complement C2	catalytic activity, acting on a protein	immune system process
C9JB90	RAB6B, member RAS oncogene family	gtpase activity	vesicle-mediated transport
F8WF65	Elongation factor 1-beta		
CON__P08727	Keratin, type I cytoskeletal 19	structural molecule activity	anatomical structure development
D6RC26	Mitogen-activated protein kinase 10		
D6RFG5	Annexin	lipid binding	
E7EMC6	Annexin	lipid binding	anatomical structure development
E7EQR4			
E9PIT3	Prothrombin	hydrolase activity	wound healing
E9PKG6	Nucleobindin 2		
H7C144	Alpha-actinin-4	cytoskeletal protein binding	cytoskeleton organization
F8VQY6	Large ribosomal subunit protein uL10	rna binding	cytoplasmic translation
F8WBR5	Calmodulin 2		muscle system process
G5E9U8	Poly (ADP-ribose) polymerase family, member 9, isoform CRA_b	transferase activity	immune system process
H0Y704	Zinc finger protein 185 with LIM domain		
I3L397	Eukaryotic translation initiation factor 5A	translation regulator activity	
J3KPA1	Cysteine rich secretory protein 3		
J3QRP6	Na(+)/H(+) exchange regulatory cofactor NHE-RF1	molecular adaptor activity	protein localization to plasma membrane
K7EMR1	Granulin precursor		inflammatory response
K7ELL7	Glucosidase 2 subunit beta	rna binding	anatomical structure development
M0QX47	Glia maturation factor gamma		cytoskeleton organization
O43866	CD5 antigen-like	hydrolase activity	immune system process
O95171-3	Sciellin		anatomical structure development
Q5SRP5	Apolipoprotein M	transporter activity	protein-containing complex assembly
P00739	Haptoglobin-related protein	catalytic activity, acting on a protein	
P00747	Plasminogen	catalytic activity, acting on a protein	wound healing
P01040	Cystatin-A	catalytic activity, acting on a protein	cell adhesion
P01591	Immunoglobulin J chain	molecular adaptor activity	immune system process
P01594	Immunoglobulin kappa variable 1–33		immune system process
P01742	Immunoglobulin heavy variable 1–69		immune system process
P02042	Hemoglobin subunit delta	oxidoreductase activity	
P02549-2	Spectrin alpha chain, erythrocytic 1	structural molecule activity	cytoskeleton organization
P02748	Complement component C9		immune system process
P02749	Beta-2-glycoprotein 1	lipid binding	lipid metabolic process
P04003	C4b-binding protein alpha chain	rna binding	immune system process
P04040	Catalase	antioxidant activity	programmed cell death
P04196	Histidine-rich glycoprotein	molecular function regulator activity	wound healing
P06727	Apolipoprotein A-IV	lipid binding	lipid metabolic process
P06870-2	Kallikrein-1	catalytic activity, acting on a protein	circulatory system process
P07476	Involucrin		cell differentiation
P09228	Cystatin-SA	hydrolase activity	nervous system process
P09382	Galectin-1	rna binding	immune system process
P10909-4	Clusterin	cytoskeletal protein binding	immune system process
P11215	Integrin alpha-M	isomerase activity	immune system process
P14174	Macrophage migration inhibitory factor	isomerase activity	immune system process
P14923	Junction plakoglobin	molecular adaptor activity	anatomical structure development
P15515	Histatin-1		anatomical structure development
P16403	Histone H1.2	dna binding	dna recombination
P18510-4	Interleukin-1 receptor antagonist protein	molecular transducer activity	inflammatory response
P20160	Azurocidin	catalytic activity, acting on a protein	immune system process
P22528	Cornifin-B	structural molecule activity	anatomical structure development
P23280-2	Carbonic anhydrase 6	lyase activity	nervous system process
P26641	Elongation factor 1-gamma	translation regulator activity	
P27169	Serum paraoxonase/arylesterase 1	hydrolase activity	lipid metabolic process
P30048-2	Thioredoxin-dependent peroxide reductase, mitochondrial	oxidoreductase activity	programmed cell death
P30086	Phosphatidylethanolamine-binding protein 1	molecular function regulator activity	signaling
P30740	Leukocyte elastase inhibitor	hydrolase activity	
P31025	Lipocalin-1	hydrolase activity	nervous system process
Q5SX91	Rab GDP dissociation inhibitor	gtpase activity	signaling
P32320	Cytidine deaminase	hydrolase activity	nucleobase-containing small molecule metabolic process
P32926	Desmoglein-3		cell adhesion
P35321	Cornifin-A	structural molecule activity	anatomical structure development
P35326	Small proline-rich protein 2A	lipid binding	immune system process
P36952-2	Serpin B5	molecular function regulator activity	anatomical structure development
X6RJP6	Transgelin 2	cytoskeletal protein binding	cytoskeleton organization
P41218	Myeloid cell nuclear differentiation antigen	dna binding	immune system process
P43652	Afamin		
P60903	Protein S100-A10	gtpase activity	anatomical structure development
P60953	Cell division control protein 42 homolog	gtpase activity	anatomical structure development
P61158	Actin-related protein 3	structural molecule activity	defense response to other organism
P61981	14-3-3 protein gamma	transferase activity	signaling
Q5JR95	Small ribosomal subunit protein eS8	structural molecule activity	ribosome biogenesis
P62701	Small ribosomal subunit protein eS4, X isoform	rna binding	cytoplasmic translation
P62857	Small ribosomal subunit protein eS28	structural molecule activity	ribosome biogenesis
Q01469	Fatty acid-binding protein 5	lipid binding	signaling
Q01518	Adenylyl cyclase-associated protein 1	cytoskeletal protein binding	cytoskeleton organization
Q04917	14-3-3 protein eta	transporter activity	signaling
Q08999	Retinoblastoma-like protein 2	transferase activity	regulation of dna-templated transcription
Q15084-3	Protein disulfide-isomerase A6	catalytic activity, acting on a protein	protein folding
Q6P4A8	Phospholipase B-like 1	hydrolase activity	lipid metabolic process
Q7Z333-3	Probable helicase senataxin	dna binding	regulation of dna-templated transcription
Q7Z6B7	SLIT-ROBO Rho GTPase-activating protein 1	gtpase activity	signaling
Q8IYF3-2	Testis-expressed protein 11		programmed cell death
Q8N4F0	BPI fold-containing family B member 2	lipid binding	
Q8TAX7	Mucin-7	immune system process	
Q93084-4	Sarcoplasmic/endoplasmic reticulum calcium ATPase 3	molecular function regulator activity	transmembrane transport
Q96FQ6	Protein S100-A16	rna binding	
Q96PD5	N-acetylmuramoyl-L-alanine amidase	hydrolase activity	immune system process
Q9H4B7	Tubulin beta-1 chain	gtpase activity	cytoskeleton organization
Q9HCY8	Protein S100-A14		immune system process
Q9NZT1	Calmodulin-like protein 5	catalytic activity	anatomical structure development
Q9UBC9	Small proline-rich protein 3	structural molecule activity	anatomical structure development
**Desplac—3 months**			
C9JHS9	High density lipoprotein binding protein		
A0A087WTS8			
B7Z8D3	Proteasome activator subunit 3	molecular function regulator activity	protein catabolic process
F2Z2I8	Stomatin like 2	mitochondrion organization	
B4DDC6	Prostaglandin E synthase 3	isomerase activity	lipid metabolic process
A0A087X0X3			
A0A087X1I3			
A0A0B4J1V0	Immunoglobulin heavy variable 3–15		immune system process
K7EM73	Calpain small subunit 1		
A0A0D9SEI8	Y-box binding protein 3		
A0A2R8YDH3	RNA helicase	hydrolase activity	reproductive process
F8VNW6	Prefoldin subunit 5		
D6R991	Matrin 3		
A0A0U1RQV5	Eukaryotic translation initiation factor 6	translation regulator activity	ribosome biogenesis
A0A286YFA2	Phosphoglycerate dehydrogenase	oxidoreductase activity	amino acid metabolic process
A0A2R8YG21	Hydroxyacyl-CoA dehydrogenase trifunctional multienzyme complex subunit alpha	catalytic activity	lipid metabolic process
A0A494C1T2	C-1-tetrahydrofolate synthase, cytoplasmic	oxidoreductase activity	cellular modified amino acid metabolic process
A0A5F9ZHL7	Acetyl-CoA acetyltransferase 1	transferase activity	lipid metabolic process
A0A5F9ZHM4	L-lactate dehydrogenase	oxidoreductase activity	
A0A7I2YQB2	Stress-70 protein, mitochondrial	Protein folding chaperone	
H7C1J8	Heterogeneous nuclear ribonucleoprotein A3	rna binding	mrna metabolic process
A0A7I2V3E1	Poly [ADP-ribose] polymerase	transferase activity	immune system process
A0A7I2V4K9	Non-POU domain containing octamer-binding	rna binding	regulation of dna-templated transcription
A0A7I2V5S2	Nucleophosmin	rna binding	ribosome biogenesis
B4DEH5	Leukotriene A4 hydrolase	hydrolase activity	
B5MD47	Septin 2	gtpase activity	vesicle-mediated transport
C9J4Z3	Ribosomal protein L37a	structural molecule activity	
C9JI87	Voltage-dependent anion-selective channel protein 1	transporter activity	transmembrane transport
C9JIZ6			
C9JTK6	Obg like ATPase 1	hydrolase activity	
C9JZ20	Prohibitin		mitochondrion organization
E7ENZ3	Chaperonin containing TCP1 subunit 5	protein folding chaperone	
E9PRI2	Eukaryotic translation initiation factor 3 subunit M		
E9PCY7	Heterogeneous nuclear ribonucleoprotein H1	rna binding	
E9PEX6	Dihydrolipoyl dehydrogenase	oxidoreductase activity	
E9PHT9	Annexin	lipid binding	
F5H5D3	Tubulin alpha chain	structural molecule activity	cytoskeleton organization
F8VR69	Ribosomal protein L6	structural molecule activity	cytoplasmic translation
F8VR82	Serine/threonine-protein phosphatase	hydrolase activity	generation of precursor metabolites and energy
F8W0G4	Poly(rC) binding protein 2	dna binding	
F8WAM2	T-complex protein 1 subunit eta	hydrolase activity	
G3V279	Enhancer of rudimentary homolog		
H0YHG0	DnaJ homolog subfamily C member 14		
H0YL83	Electron transfer flavoprotein subunit alpha		lipid metabolic process
J3KMX5	Small ribosomal subunit protein uS15	Small ribosomal subunit protein uS15	
J3QR48	Importin subunit beta-1	molecular carrier activity	nucleocytoplasmic transport
J3QT56	Huntingtin interacting protein K		programmed cell death
O00244	Copper transport protein ATOX1	molecular carrier activity	
Q32Q12	Nucleoside diphosphate kinase	transferase activity	nucleobase-containing small molecule metabolic process
O75531	Barrier-to-autointegration factor	dna binding	chromatin organization
O75607	Nucleoplasmin-3	histone binding	chromatin organization
O75937	DnaJ homolog subfamily C member 8		
P00505	Aspartate aminotransferase, mitochondrial	transferase activity	amino acid metabolic process
P18754	Regulator of chromosome condensation	molecular function regulator activity	mitotic cell cycle
P23246	Splicing factor, proline- and glutamine-rich	dna binding	regulation of dna-templated transcription
P25398	Small ribosomal subunit protein eS12	structural molecule activity	cytoplasmic translation
P25705-2	ATP synthase subunit alpha, mitochondrial	atp-dependent activity	generation of precursor metabolites and energy
P27348	14-3-3 protein theta		signaling
P30049	ATP synthase subunit delta, mitochondrial	transporter activity	generation of precursor metabolites and energy
P31946-2	14-3-3 protein beta/alpha	molecular function regulator activity	signaling
P32119	Peroxiredoxin-2	antioxidant activity	immune system process
P40926-2	Malate dehydrogenase, mitochondrial	oxidoreductase activity	oxidoreductase activity
P45973	Chromobox protein homolog 5	histone binding	regulation of dna-templated transcription
P46783	Small ribosomal subunit protein eS10	structural molecule activity	cytoplasmic translation
P49321-4	Nuclear autoantigenic sperm protein	histone binding	chromatin organization
P50991-2	T-complex protein 1 subunit delta	protein folding chaperone	protein folding
P53396-3	ATP-citrate synthase	transferase activity	lipid metabolic process
P54727-2	UV excision repair protein RAD23 homolog B	dna binding	protein catabolic process
P62258	14-3-3 protein epsilon	transporter activity	signaling
P62995-3	Transformer-2 protein homolog beta	rna binding	mrna metabolic process
P78371-2	T-complex protein 1 subunit beta	protein folding chaperone	protein folding
Q00688	Peptidyl-prolyl cis-trans isomerase FKBP3	rna binding	
Q08211	ATP-dependent RNA helicase A	atp-dependent activity	regulation of dna-templated transcription
Q13155	Aminoacyl tRNA synthase complex-interacting multifunctional protein 2	molecular adaptor activity	programmed cell death
Q8WUM4	Programmed cell death 6-interacting protein		programmed cell death
Q99714	3-hydroxyacyl-CoA dehydrogenase type-2	oxidoreductase activity	lipid metabolic process
Q9Y2X3	Nucleolar protein 58	rna binding	ribosome biogenesis
Q9Y5S9-2	RNA-binding protein 8A	rna binding	mrna metabolic process

## Data Availability

The original contributions presented in the study are included in the article/Supplementary Material, further inquiries can be directed to the corresponding authors.

## Supplementary Materials

The following supporting information can be downloaded at: https://www.mdpi.com/article/10.3390/gels10120772/s1, Table S1: Full-mouth gingival and plaque index evaluation of the study population at baseline and after 3 months according to scores. Proportion (%); Table S2: Percentage (%) of sites with bleeding on probing.
