# Supplementary material for: The Effect of a Nature-Based Gel on Gingival Inflammation and the Proteomic Profile of Crevicular Fluid: A Randomized Clinical Trial
Source: Gels. 2024 Nov 27;10(12):772. doi: 10.3390/gels10120772 (PMC11675388; doi:10.3390/gels10120772)
Supplement: Supplementary file 1 [file gels-10-00772-s001.zip › gels-3294129-supplementary.pdf]

gels MDPI

Supplemental material

Effect of a Natural-Based Gel on gingival inflammation and Proteomic Profile of crevicular fluid: A Randomized Clinical Trial

Luciene Cristina Figueiredo[1], Bruno Bueno-Silva[1,2], Giovanna Denúncio[1], Nathalia Freitas Figueiredo[1], Daniele Ferreira da Cruz[1], Jamil A. Shibli[1], Maria Helena R. Borges[3], Valentim A. R. Barão[3], Doron Haim[4], Thabet Asbi[1,4,5*], João Gabriel S. Souza[1*]

Table S1 - Full-mouth gingival and plaque index evaluation of the study population at baseline and after 3 months according to scores. Proportion (%).

Variable	Score	Time	Desplac	Oral B
Plaque index %	0	Base	30%	29%
		3 months	54%	45%
	1	Base	59%	53%
		3 months	40%	47%
	2	Base	11%	18%
		3 months	5%	7%
	3	Base	0%	0%
		3 months	1%	1%
Gingival index %	0	Base	30%	31%
		3 months	51%	44%
	1	Base	61%	55%
		3 months	47%	51%
	2	Base	9%	14%
		3 months	2%	5%
	3	Base	0%	0%
		3 months	0%	0%

Table S2 – Percentage (%) of sites with bleeding on probing.

Group	Baseline	3 months
DESPLAC	69%	72%
Oral B	71%	58%
